# Supplementary material for: Comparison of perceived family state and functioning among individuals with depression and general population in Southern Thailand
Source: PLoS One. 2025 May 16;20(5):e0323866. doi: 10.1371/journal.pone.0323866 (PMC12083788; doi:10.1371/journal.pone.0323866)
Supplement: Attachment — Submitted filename: PONE-D-24-52399 - revision.docx [file pone.0323866.s001.docx]

**Major Revisions**

**Introduction and clarity of hypotheses**

- The introduction does not clearly delineate how this study advances knowledge. A more explicit discussion of gaps in prior Thai and international research is needed.
- The hypotheses are not clearly stated as testable assertions. The manuscript should include explicit statements.
- The study does not specify which dimensions of family functioning (e.g., support, discipline) are expected to differ significantly. A dedicated section on objectives and hypotheses should be added for clarity.
- The manuscript does not clearly state the sources for FSFAS-25 and PHQ-9. Are there validated Thai versions of these scales available?

**Statistical Analysis**

- The study does not employ multivariate techniques (e.g., regression, ANCOVA) to adjust for demographic variables such as income and education, which may confound the relationship between depression and family functioning. Without a multivariate model (e.g., multiple regression or ANCOVA), it is impossible to determine whether the observed differences in family functioning scores are truly due to depression or are confounded by socioeconomic factors.
- The arbitrary cutoff (≥9 vs. <9) oversimplifies depressive symptoms, and the small residual depression subgroup (n=16) reduces statistical power, increasing the risk of Type II errors. The threshold of 9, while clinically valid, may oversimplify the continuum of depressive symptoms. A more nuanced analysis (e.g., treating PHQ-9 as a continuous variable) would provide better insight.
- Tests such as the t-test and Chi-square assume normality of data distribution and sufficient sample sizes per category. For small groups (e.g., n=16 in the residual depression subgroup), these assumptions may be violated, leading to unreliable p-values. Non-parametric alternatives (e.g., Mann-Whitney U test) are more appropriate but underpowered for small samples. While the Shapiro-Wilk test for normality is mentioned, the results are not reported in the manuscript, leaving uncertainty about whether appropriate tests were chosen for the data.
- The manuscript emphasizes significant findings (e.g., family support differences) without adequately addressing the effect sizes. Reporting effect sizes alongside p-values would help readers understand the practical significance of the observed differences.

**Discussion Section**

- Statements about family functioning’s role in depression recovery are too broad given methodological limitations. Conclusions should be framed more cautiously. The small sample size (41 individuals per group) obtained through convenience sampling significantly limits the generalizability of findings. Furthermore, the division of the sample into subgroups (PHQ-9 ≥9 vs. <9) compounds the problem, as the statistical power decreases substantially when analyzing smaller subgroups. These limitations should have been explicitly addressed in the discussion and considered in the design stage.
- The discussion relies heavily on Thai studies, limiting global relevance. A broader literature review is necessary.
- The possibility that depressive symptoms distort self-perceptions of family functioning should be addressed. Consideration of physical illness and socioeconomic confounders is also necessary.
- Recommendations for family-focused interventions are vague. The manuscript should specify evidence-based approaches that could be implemented in practice.

**Minor Revisions**

**Language and Clarity**

- The manuscript contains errors and awkward phrasing. Examples:
  - *"compare these perceptions to those of the general population"* → should be *"compare these perceptions with those of the general population."*
  - *"highlighting the importance of comprehensive treatment approaches aimed at achieving complete symptom remission"* → should be revised for conciseness.
- Improve clarity and consistency in reporting statistical findings.

**References**

- Several references (e.g., Lines 304–312) should be updated with recent studies on family dynamics and depression.
- Ensure all citations follow the required journal style.
