# Supplementary material for: Comparison of perceived family state and functioning among individuals with depression and general population in Southern Thailand
Source: PLoS One. 2025 May 16;20(5):e0323866. doi: 10.1371/journal.pone.0323866 (PMC12083788; doi:10.1371/journal.pone.0323866)
Supplement: Attachment — Submitted filename: response to reviewer.docx [file pone.0323866.s003.docx]

**Major Revisions**

**Introduction and clarity of hypotheses**

- The introduction does not clearly delineate how this study advances knowledge. A more explicit discussion of gaps in prior Thai and international research is needed.
- *Response: Thanks, we have added more information as suggest “Most existing studies have focused on Western contexts, which may overlook the unique complexities of Thai families, which are influenced by cultural, social, and religious factors, including Buddhism and Islam. While Buddhism predominantly influences the cultural context in Thailand, the southern region is distinct due to its significant Muslim population. This multicultural and multi-religious composition may lead to variations in family dynamics compared to other regions and Western countries. Additionally, in Thailand, there has been only one study comparing family functioning in families of psychiatric patients to nonclinical families [22]. No previous studies have focused on the families of individuals with MDD.”*
- The hypotheses are not clearly stated as testable assertions. The manuscript should include explicit statements.
- *Response:* *Thanks, we have added more information as suggest “Family functioning refers to…… [20-21]. Studies have shown that families of individuals with psychiatric disorders often experience dysfunction in areas such as problem-solving, emotional responsiveness, involvement, communication, and behavior control [22]. ……………………. While family functioning among individuals with psychiatric disorders tends to be less healthy than the general population [22], many Thai individuals with MDD continue to experience residual symptoms [7]. Therefore, family dysfunction, such as poor problem-solving and low emotional responsiveness, may be factors contributing to residual depression [24].*
- The study does not specify which dimensions of family functioning (e.g., support, discipline) are expected to differ significantly. A dedicated section on objectives and hypotheses should be added for clarity.
- *Response: Thanks, we have added more information as suggest in the part of introduction and objective as “This study aims to 1) explore the characteristics of family functioning (including family support, discipline, communication, problem-solving, emotional status, and relationships) and 2) compare family functioning between individuals with MDD and the general population.”*
- The manuscript does not clearly state the sources for FSFAS-25 and PHQ-9. Are there validated Thai versions of these scales available?
- *Response: Thanks, we have added* *more information as “This is a self-report questionnaire designed to evaluate family state and functioning assessment scale (FSFAS) [26] that is mainly based on the structural domains of the Family Assessment Device from the McMaster Model of Family Functioning (MMFF) [27], Chulalongkorn family inventory [28], and the Thai family functioning scale [29].” and calculate more “ Cronbach's alpha coefficient of 0.94 for the total, and 0.72 - 0.91 for the subscales,” using data from our study’*

**Statistical Analysis**

- The study does not employ multivariate techniques (e.g., regression, ANCOVA) to adjust for demographic variables such as income and education, which may confound the relationship between depression and family functioning. Without a multivariate model (e.g., multiple regression or ANCOVA), it is impossible to determine whether the observed differences in family functioning scores are truly due to depression or are confounded by socioeconomic factors.
- Tests such as the t-test and Chi-square assume normality of data distribution and sufficient sample sizes per category. For small groups (e.g., n=16 in the residual depression subgroup), these assumptions may be violated, leading to unreliable p-values. Non-parametric alternatives (e.g., Mann-Whitney U test) are more appropriate but underpowered for small samples. While the Shapiro-Wilk test for normality is mentioned, the results are not reported in the manuscript, leaving uncertainty about whether appropriate tests were chosen for the data.
- The manuscript emphasizes significant findings (e.g., family support differences) without adequately addressing the effect sizes. Reporting effect sizes alongside p-values would help readers understand the practical significance of the observed differences.
- *Response: I sincerely apologize that my previous manuscript caused misunderstandings. Because our study aims to compare perceived family state and functioning among individuals with depression and general population, it is not a relationship study. Therefore, we revised the manuscript to be more accurate and add more information as “Variables with a p-value > 0.05 were considered normally distributed, while those with a p-value ≤ 0.05 were considered non-normally distributed. Based on these results, appropriate parametric or non-parametric tests were selected for further analysis.”*
- *We calculate additional statistical values such as Fisher's exact test p-value in Table 3.*
- *Thank you very much to the reviewer for making us see this error.*
- The arbitrary cutoff (≥9 vs. <9) oversimplifies depressive symptoms, and the small residual depression subgroup (n=16) reduces statistical power, increasing the risk of Type II errors. The threshold of 9, while clinically valid, may oversimplify the continuum of depressive symptoms. A more nuanced analysis (e.g., treating PHQ-9 as a continuous variable) would provide better insight.
- *Response: we have added this point as limitation* *“However, our study found no significant statistical difference in the communication and problem-solving dimensions between the MDD group and the general population (p=0.087), as well as between the residual and non-residual MDD groups (p=0.074). This lack of difference may be attributed to the small sample size limitation. Future studies should involve a larger population to further explore these dimensions.”*

**Discussion Section**

- Statements about family functioning’s role in depression recovery are too broad given methodological limitations. Conclusions should be framed more cautiously. The small sample size (41 individuals per group) obtained through convenience sampling significantly limits the generalizability of findings. Furthermore, the division of the sample into subgroups (PHQ-9 ≥9 vs. <9) compounds the problem, as the statistical power decreases substantially when analyzing smaller subgroups. These limitations should have been explicitly addressed in the discussion and considered in the design stage.
- The discussion relies heavily on Thai studies, limiting global relevance. A broader literature review is necessary.
- The possibility that depressive symptoms distort self-perceptions of family functioning should be addressed. Consideration of physical illness and socioeconomic confounders is also necessary.
- Recommendations for family-focused interventions are vague. The manuscript should specify evidence-based approaches that could be implemented in practice.
- *Response: Thanks, we have added this point as limitation such as*

*“This approach may introduce biases, as some participants might report negative perceptions of family support influenced by their symptoms of depression.”*

*‘Cultural and regional differences could influence family dynamics, so generalizing the findings to other regions or countries may not be entirely appropriate without further study.’*

*“As a result, physical illness, education level, and socioeconomic factors should be considered potential confounders.”*

*“designing a prospective therapeutic study that includes family interventions targeting specific aspects of family functioning, such as family support, discipline, and emotional status, along with long-term follow-ups, could provide valuable insights for treatment planning and enhance understanding of family dynamics in MDD.”*

- Recommendations for family-focused interventions are vague. The manuscript should specify evidence-based approaches that could be implemented in practice.
- *We tried to revised the discussion and conclusion as “Individuals with depression demonstrate lower family functioning compared with the general population. To improve treatment outcomes, healthcare providers should implement strategies that enhance family functioning, particularly in areas like family support and emotional well-being. A holistic approach that addresses these aspects can promote better emotional health, strengthen family relationships, and support recovery.”*
- ***We would like to thank the reviewers for your kind advice and help in making this manuscript more academically valuable. And we hope to receive kindness from the honorable view again.***

**Minor Revisions**

**Language and Clarity**

- The manuscript contains errors and awkward phrasing. Examples:
  - *"compare these perceptions to those of the general population"* → should be *"compare these perceptions with those of the general population."*
  - *"highlighting the importance of comprehensive treatment approaches aimed at achieving complete symptom remission"* → should be revised for conciseness.
- Improve clarity and consistency in reporting statistical findings.

*Response: We tried to corrected this manuscript and has been approved by* *the Office of International Affairs, Faculty of Medicine, Prince of Songkla University.* *If the reviewer has any additional suggestions, please feel free to inform us.*

**References**

- Several references (e.g., Lines 304–312) should be updated with recent studies on family dynamics and depression.
- Ensure all citations follow the required journal style.

*Response:* *we would like to thank the reviewers for your kind advice and help in making this manuscript more academically valuable. And we hope to receive kindness from the honorable view again.*
