# Supplementary material for: Comparison of perceived family state and functioning among individuals with depression and general population in Southern Thailand
Source: PLoS One. 2025 May 16;20(5):e0323866. doi: 10.1371/journal.pone.0323866 (PMC12083788; doi:10.1371/journal.pone.0323866)
Supplement: Attachment — Submitted filename: response_to_reviewer_auresp_2.docx [file pone.0323866.s004.docx]

Point to point response

Editors

Please review your reference list to ensure that it is complete and correct. If you have cited papers that have been retracted, please include the rationale for doing so in the manuscript text, or remove these references and replace them with relevant current references. Any changes to the reference list should be mentioned in the rebuttal letter that accompanies your revised manuscript. If you need to cite a retracted article, indicate the article’s retracted status in the References list and also include a citation and full reference for the retraction notice.

*Response: We have reviewed and removed references 17 and 19, and have replaced reference 22 with more relevant and current sources (the new reference series is now numbered as ref. 20).*

*Thank you so much for your valuable suggestions, which have made our manuscript more scientifically rigorous*.

Reviewer #1:

The authors have provided a more detailed discussion of gaps in prior research, particularly within the Thai context, emphasizing the influence of cultural and religious factors on family dynamics. While they have formulated clearer and more testable hypotheses, the specific dimensions of family functioning expected to differ (e.g., support, discipline) are not explicitly outlined.

*Response: Thank you for your valuable suggestions. We have included more information about the characteristics of Muslim families: “A Muslim family is often characterized by strong unity and implements love, cooperation, respect and mutual understanding between each other [23]. This multicultural and multi-religious composition may lead to variations in family dynamics compared to both other regions within Thailand as well as in Western countries”*

At the methodological level, the authors' approach has several strengths but also notable limitations that may impact the validity and generalizability of the results. A key concern is the absence of effect sizes, which limits readers' ability to assess the practical significance of the findings. Although the sample size was calculated to ensure statistical power, it remains relatively small, particularly when divided into subgroups (e.g., those with PHQ-9 ≥9 vs. <9). This reduction in sample size diminishes statistical power and increases the risk of type II errors (i.e., failing to detect true differences). For instance, the subgroup with residual symptoms (PHQ-9 ≥9) comprises only 16 participants, which restricts the generalizability of the findings. To enhance the study's robustness, future research could consider a longitudinal design, a larger and more diverse sample, and the application of more advanced statistical techniques.

*Response: Thank you for your valuable concern regarding the use of multivariate techniques to adjust for confounders. We acknowledge the importance of adjusting for potential confounding factors to ensure the robustness of our findings.*

*Our decision not to use multivariate techniques was driven by the primary objective of our study, which was to examine perceived family functioning among individuals with MDD and compare it to that of the general population, rather than identifying associated factors. Given this focus, our analysis centered on direct group comparisons without adjusting for confounders. However, we recognize that the lack of such adjustments may influence the magnitude and directionality of the observed differences.*

*To address this, we have revised our discussion to explicitly acknowledge this limitation and its potential impact on our findings. While our conclusions remain valid, we will refine our recommendations to emphasize the need for further research incorporating multivariate approaches to explore the role of potential confounders in greater depth.*

*We appreciate this valuable feedback and will ensure that our revised manuscript provides a clearer rationale for our methodological choices and their implications*

The authors have appropriately moderated their conclusions and acknowledged the study's limitations, such as the small sample size and reliance on convenience sampling. They have also expanded the discussion to include international studies, though the literature review remains predominantly focused on Thai research. Additionally, they have addressed the potential for depressive symptoms to distort self-perceptions of family functioning, which strengthens the manuscript's critical perspective.

*Response: Thank you for your appreciation.*

Answer to Reviewer 1:

For the comment regarding "the hypothesis are not clearly stated": The aims of the study were mentioned, yet, they seems incomplete as they failed to mention the aim that patients with MDD with residual symptoms would be compared to those without residual symptoms.

*Response: Thank you for your valuable suggestions. We have added the mentioned aim: “Additionally, since some individuals with MDD may experience residual depression symptoms, this study also compared the family functioning characteristics of those with and without these residual depression symptoms.”*

The manuscript now describes the dimensions of the construct family functioning, yet for those not familiar with the scale, we cannot tell if each of the 5 domains have the same weight in the overall score (do all 5 domains contribute with the same number of questions?). Also, regarding this variable, only information on the scale range is provided, but for results in table 2 and 3, the results for family functioning (overall and its different components) were dichotomized in low or moderate versus high family support (for example). Which cut-off was used to create these groups and why? Please add that info in the method section.

*Response: Thank you for your valuable suggestions. We have added more information as The scale is organized into five dimensions: family support (items 1-5), discipline (items 6-11), communication and problem-solving (items 12-16), emotional status (items 17-21), and relationships (items 22-25). It is classified into high, moderate, and low levels.*

|  | *Family functioning levels* | | |
| --- | --- | --- | --- |
|  | *Low* | *Moderate* | *High* |
| *Family support* | *5-9* | *10-14* | *15 and above* |
| *Discipline* | *6-11* | *12-17* | *18 and above* |
| *Communication and problem solving* | *5-9* | *10-14* | *15 and above* |
| *Emotional status* | *5-9* | *10-14* | *15 and above* |
| *Relationship* | *4-7* | *8-11* | *12 and above* |
| *Total* | *25-49* | *50-74* | *75 and above* |

Regarding the statistical analyses, reviewer question why authors did not use multivariate techniques to adjust for confounders. I believe that the acknowledgement that those factors were confounders in the discussion is not sufficient to address the reviewer's comment. Since you have information on the variables that were potential confounders, it is unclear why you did not assess the role of those factors in the comparisons performed. Also, there is not discussion on what the is the potential impact on the magnitude and directionality of the results because of the "lack of adjustment" for those factors. If there is a reasons justifying why that adjustment was not done, it was not presented in the discussion. Thus, the conclusions and recommendations needs to adjusted accordingly.

*Response: Thank you for your valuable concern regarding the use of multivariate techniques to adjust for confounders. We acknowledge the importance of adjusting for potential confounding factors to ensure the robustness of our findings.*

*Our decision not to use multivariate techniques was driven by the primary objective of our study, which was to examine perceived family functioning among individuals with MDD and compare it to that of the general population, rather than identifying associated factors. Given this focus, our analysis centered on direct group comparisons without adjusting for confounders. However, we recognize that the lack of such adjustments may influence the magnitude and directionality of the observed differences.*

*To address this, we have revised our discussion to explicitly acknowledge this limitation and its potential impact on our findings. While our conclusions remain valid, we will refine our recommendations to emphasize the need for further research incorporating multivariate approaches to explore the role of potential confounders in greater depth.*

On a extra note: Method section would benefit from having further details on sample size calculation (power and alpha error estimated and expected "effect size" should be mentioned. Also, regarding sample selection, further details on how many cases of MDD / community controls were contacted and how many accepted to participate (the response "rate") should be provided so that readers can assess potential for selection biases risks.

*Response: Thank you for your suggestion, we have revised* *the details on sample size calculation as “ A power analysis was performed to calculate the required sample size to compare the proportion of dysfunctional families between the two groups: individuals with MDD and the general population. The analysis was based on the following parameters: a significance level of 0.05, a power of 0.80, and expected proportions of dysfunctional families of 83.3% in the individuals with MDD group, and 45.0% in the general population group [24]. Assuming equal group sizes (n2/n1 = 1), the analysis estimated that 29 participants per group (total 58) would be required to detect a significant difference. To ensure data robustness, and account for potential participant drop-out, the sample size was increased to 82 participants (41 per group).”*

Discussion section: The point proposed by the reviewer on possibility of depressive symptoms distorting self-perception of family function in those with MDD is likely the biggest limitation of the present manuscript and needs to be discussed in greater depth. The present study does not allow teasing apart temporality of the association between MDD and lower family functioning (whether depression was the cause of the patient reporting lower family functioning or if lower family functioning lead to MDD or MDD with worse control). Consider discussing findings from prospective studies to provide an idea of the potential impact of such limitation on the findings. If recall bias and/or reverse causality biases are the main reason for the findings hereby reported, then the implications are that the study findings should be taken in light of those limitations and possibly as major implications they should propose future prospective studies. The point is that the implications now are too strong as if there were no major issues in the interpretation of the findings hereby presented.

*Response: Thank you for your suggestion, we have revised as* *“Furthermore, the primary objective of our study was to examine perceived family functioning among individuals with MDD and to compare it with that of the general population, rather than identify associated factors. Consequently, our analysis focused on direct group comparisons without adjusting for confounders. However, the absence of adjustments may influence the magnitude and direction of the observed differences, potentially leading to over- or underestimation of family functioning differences between the groups.*

*Finally, future studies should focus on a longitudinal design, involve a larger and more diverse population, and use a representative sample, ideally employing a multi-center approach and including participants from various regions of Thailand. ………. Applying more advanced statistical techniques; including multivariate approaches, to explore the role of potential confounders in greater depth could enhance the understanding of family dynamics in MDD.”*

*Finally, thank you for your kindness and patience in advising us, as our manuscript look more scientifically valuable.*

Answer to reviewer 2:

Language and clarity: Partially solved. I suggest further editorial review to improve text. Some terms used does not seem accurate.

For example, lines:

110 "They underwent training on study procedures" - this is the type of statement that applies best to data collection, or researchers, but not to participants who take surveys.

115 " the sample calculation used in the literature review". It does not make sense as sample size is not "used" for literature review purposes.

line 119 and 120 - reliability and accuracy terms used as synonyms

line 77: "residual depression". Consider keeping the wording residual depression symptoms", which would be most appropriate.

Response: *Thank you for your suggestion. We have made all the corrections, and the manuscript has been proofread and edited by Mr. Andrew Tait from the Office of International Affairs, Faculty of Medicine, Prince of Songkla University.*

*Finally, thank you for your kindness and patience in advising us, as our manuscript look more scientifically valuable.*

Reviewer #3: Thank you for taking the time to revise the manuscript and respond to the reviewers questions.

*Response: Thank you for your appreciation.*
