# Supplementary material for: Comparison of perceived family state and functioning among individuals with depression and general population in Southern Thailand
Source: PLoS One. 2025 May 16;20(5):e0323866. doi: 10.1371/journal.pone.0323866 (PMC12083788; doi:10.1371/journal.pone.0323866)
Supplement: Attachment — Submitted filename: response_to_reviewer_auresp_3.docx [file pone.0323866.s005.docx]

Response to reviewer

There are two issues that I believe deserve final adjustments.

First, there were some inconsistencies in the text regarding the FSAS score.

In line 148 it reads " FSFAS score, which ranges from 30 to 120... is computed by summing the raw scores across all 30 items,". Yet, the table added follows with inconsistent scores (ranging from the lowest value of 25). Is the range from 25 to 120, then? Also, the 5 dimension explanation in line 150-152 reads "The scale is organized into five dimensions: family support (items 1-5), discipline (items 6-11) communication and problem-solving (items 12-16), emotional status (items 17-21), and relationships (items 22-25). Where are items 26-30? Which dimension do they belong to?

*Response: We have reviewed the error in the content of questionnaires as ‘Positive statements include items 1-16, whereas negative statements consist of items 17- 25. The total FSFAS score ranges from 25 to 100 and is obtained by summing the raw scores across all 25 items, with higher scores indicating better family functioning’.*

Finally, as mentioned previously, the results might be the result of methodological limitations (ex: lack of adjustment for confounders, lack of ability to assess directionality of association (reverse-causality), generalizability. Yet, the recommendation in lines 302-303 "To improve outcomes, reduce the likelihood of residual depression symptoms, and prevent relapse, healthcare providers should implement strategies that enhance family functioning as part of their holistic care." seems too strong. You should consider that your findings might not hold once limitations are solved with other studies. Thus, as proposed before, consider "toning down, for instance, by limiting your recommendation to emphasize the need for further longitudinal studies that would account for the limitations discussed.

*Response: We have reviewed as suggestion “Recognizing and addressing the role of family functioning is essential in developing comprehensive, person-centered mental health interventions for individuals with MDD. However, this study is limited by confounding variables, the inability to determine the directionality of associations (i.e., reverse causality), and issues with generalizability. Therefore, future research should focus on specific dimensions of family functioning accompanied by long-term follow-up assessments. These interventions could provide critical insights into effective treatment planning by highlighting how family dynamics influence recovery.”*

*Thank you so much for your valuable suggestions, which have made our manuscript more scientifically rigorous.*
